# An experimental outlook on investigating temporal-scale dynamics of microbial communities

**DOI:** 10.1093/ismeco/ycag086

**Published:** 2026-04-14

**Authors:** Alanna Molly Leale, Sijmen Schoustra

**Affiliations:** Laboratory of Genetics, Wageningen University and Research, Droevendaalsesteeg 1, 6708 PB Wageningen, Gelderland, Netherlands; Microbiome Adaptation to the Changing Environment Laboratory, École Polytechnique Fédérale de Lausanne, Rte des Ronquos 86, 1950 Sion, Switzerland; Laboratory of Genetics, Wageningen University and Research, Droevendaalsesteeg 1, 6708 PB Wageningen, Gelderland, Netherlands; Department of Food Science and Nutrition, School of Agricultural Sciences, University of Zambia, 10101 Lusaka, Zambia

**Keywords:** evolutionary ecology, synthetic communities, species sorting, genotypic changes, selection, coexistence

Environments in nature harbour complex species communities [1], which are known to respond to environmental changes, such as climate change and shifts in available resources. The classical view of adaptation to changing environments focuses on individual species and their potential to survive novel environments via genetic changes, or adaptive evolution [2]. However, this commonly overlooks the necessity of considering species in the context of an entire co-evolving community, both in the short and longer term. A community can respond to an altered environment both by adjustment of relative species abundances (species sorting) and by fixing spontaneous mutations spreading in individual species, with the relative importance gradually shifting overtime from species sorting to novel mutations. Both processes are traditionally studied in isolation, meanwhile growing evidence supports that in the context of adaptive responses to change, species sorting and novel mutations should be considered simultaneously [3, 4] with the relative importance gradually shifting over time.

Because of the complexity of multispecies communities, studying their adaptive dynamics has thus far been mainly limited to theoretical approaches and model simulations [3]—with more recent emergence of experimental methods that are inspired on classical experimental evolution to study adaptive dynamics of single species [5, 6]. In experimental evolution, Muller plots are an elegant visualization commonly used to track the emergence, spread, and fate of genetic variants in evolving monocultures [6–8]. While Muller plots are mostly applied to single species, we imagine they can be extrapolated to multi-species communities. Essentially, a different colour is a particular species’ abundance, and different shades of that colour are genotypes within that species (Fig. 1B).

**Figure 1:**
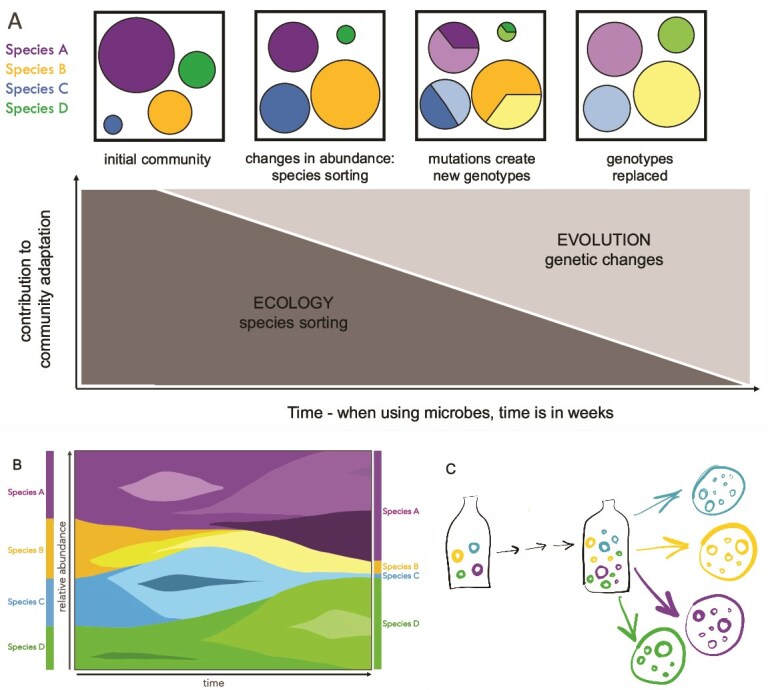
(A) a community of four species can stably co-exist in many environments. Upon a change in environmental conditions, the community may initially change in relative abundances of species (species sorting), where species intrinsically better adapted to the new conditions increase in frequency. Upon prolonged selection, novel adaptive genetic changes (mutations) may occur, further shaping the communities and changing relative species abundances. (B) Depiction of an imagined Muller-type plot of multiple species (each colour) with sub-genotypes within (shades of colours). (C) Visual depiction of propagation and selective agar plating of four species, each indicated by a unique colour. The propagation experiment would be initiated by a single isogenic clone of each species.

A core inspiration for our conceived “multi-species Muller plot” are concepts from community ecologist Mark Vellend, who proposed: can unique genotypes within a species be paralleled to different species within a community? He argues to extend the framework of population genetics to interpret community ecology, since species diversity and genetic diversity have parallel processes and patterns, but they just act at different hierarchical levels [9] (Table 1). Of further consideration is how community function compares to genotype fitness [10], such that selection could act on community members for functional traits just as genotypes with differing fitness are selected [11–13]. A community-level selection or breeding process could then arise [14, 15], where certain traits or characteristics become prevalent in a community due to selective pressures acting on collective traits, rather than on individual traits. In brief, community-level selection requires (1) variation in the community trait of interest, (2) a link between this variation and “fitness” (community function that can be selected), and (3) heritability [16, 17]. Heritability is especially critical but less conclusive at the community level—it means that if parental communities are deconstructed, then reassembled, these offspring communities will again reproduce a similar function to parental communities [15]. There is growing enthusiasm in investigating if and how selection can act at levels above individuals comprising a single species in microbial communities, and the topic is explored in detail elsewhere [10–17]. Instead in this contribution, we first define and elaborate the requirements for a model system for multi-species community experimental evolution (without explicit community level selection) and then outline some concrete research questions that can be addressed with such a system.

**Table 1:** Paralleled processes and components in population genetics and community ecology (Vellend 2016).

**Evolution (population genetics)**	**Community ecology**
Selection	Species sorting
Mutation	Speciation
Drift (genotypes)	Drift (species)
Gene flow	Dispersal (*introduction/invasion*)
Genotype	Single species
Single species	Community of species
*Species fitness*	*Community function*
*Species persistence*	*Compositional / functional stability*

Italics indicate extrapolations.

We firstly propose that an experimental model system synthetic community would have a system where members can be selectively isolated from one another, such as through selective agar plating or colony morphologies [18, 19]. Cell sorting is another potential direction to selectively separate community members [20], but cell sorters are not readily available in most labs. Furthermore, many methods exist to track absolute and relative abundances of community members, such as fluorescent tags with flow cytometry [21], trained models for untagged flow cytometry [22, 23], or advances in genetic barcoding [24, 25]. However, we and others [19] emphasize that selective isolation can effectively incorporate classic competition experiment approaches from experimental evolution [5] to multispecies adaptation, which is valuable since it permits competition experiments of evolved and ancestral members against one another. The ability to measure effects of new mutations within individual players is lacking from most current multispecies microbial community experiments. Further, more practical requirements exist. Firstly, a minimal bottleneck of complexity and comparable dynamics between natural versus laboratory communities is desired. A loss in diversity under lab conditions is a common pitfall when working with immense diversity in natural soil or gut microbiomes [26–29] where successful laboratory culturing is difficult [30, 31] and natural environmental conditions challenging to imitate. Limiting the loss of taxonomic diversity when natural communities are propagated in the lab increases the likelihood that key ecological interactions remain and simplifies studying mechanistic processes; this can be facilitated by using a natural community that contains relatively low or “manageable” diversity of key taxa and whose environment can be mimicked. Coexistence between community members should exist under the natural conditions imitated in the laboratory setup [19]. Engineering coexistence can be elegantly achieved [32–35]; however, it is not sought in this case because it limits the relevancy to nature and typically restricts the synthetic community to only a couple species. Using more natural systems can help link these approaches to other fields, such as community ecology and applied practices (food fermentation, bioremediation, etc.). We propose a requirement of stable coexistence because our objectives concentrate on studying the long-term ecological and evolutionary dynamics between community members. Initial coexistences may break down with evolutionary genetic changes, and this is precisely such outcomes that are valuable to experimentally investigate with a tractable synthetic community. Secondly, we however still suggest that the laboratory media be defined and modifiable yet reflect the natural substrate and environment. A defined manipulatable media is a powerful tool to exert selection pressures through changing available substrates and observe consequent community responses. Thirdly, experiments with the synthetic community can ideally also be performed in a natural environment, either in the natural substrate in the lab, or the natural substrate and natural “field” environment. For example, microbial communities of fermented foods meet our aforementioned requirements, as they contain a manageable level of diversity, their environments are easily reproduced in the lab (milk, grape must, tea, etc.) with reduced bottleneck in diversity, and their substrates are well studied with some already developed synthetic medias (e.g. synthetic wine must) [36–38]. Fourthly, we encourage including both bacterial and eukaryote species. Most current experimental microbial communities use only bacterial members (Table 2) [39–42], yet eukaryotic microorganisms (yeasts, fungi, protozoa, algae, viruses, etc.) are also components of many natural microbial ecosystems [37, 38, 43–45]. The combination of prokaryotes and eukaryotes in a synthetic community would be a unique and valuable aspect, which we consider in our proposed fermented food synthetic community (Box 1), though investigating the role of bacteriophages is a potential addition to include later on, and has applied relevance for fermented food production [45]. Finally, the generation time of the community to reach carrying capacity after propagation should be short. For instance, if generation time is three days, it permits 90 transfers in 1 year, or ~800 generations, which is suitable to most research project timelines.

**Table 2:** Comparison of some existing synthetic microbial community model systems.

**Paper(s)**	**# Species**	**# Transfers/generations**	**Transfer time**	**Media**	**Environment resembled**	**Members**
Cairns et al. (2018) Cairns et al. (2020)	33–34	12 cycles ~54 gen.	4 days (at 10%)	Nutrient rich (PPY), low nutrient (M9 + 1% KB)	Not mentioned	Bacteria
Piccardi et al. (2019) Piccardi et al. (2022) Piccardi et al. (2024)	4	44 cycles ~300 gen.	7 days	Metal working fluids (undefined)	Bioremediation	Bacteria
Goldford et al. (2018) Chang et al. (2023)	12	12 cycles ~84 gen.	2 days	M9/M9-glucose	Plant leaf and soil	Bacteria
Weiß et al. (2023) Weiß et al. (2022)	12	4 cycles	1 day	AF media + modified versions	Gut microbiota	Bacteria
(a) O’Brien et al. (2023) (b) Rivett et al. (2016) (c) Scheuerl et al. (2020)	(a) 2–15 (b) 1–16 (c) 22	(a) 1 cycle (b) 7 cycles (c) 22 cycles	7 days (at 10%)	(a) Iron-limited LB (b) and (c) Boiled leaf tea media (undefined)	Water filled beech tree holes	Bacteria
Pourcelot et al. (2025)	6	1 cycle	3 to 7 days	Synthetic grape must	Grape wine must	Yeast
Ruiz et al. (2023)	60	1 cycle	3 to 7 days	Synthetic grape must	Grape wine must	Yeast
(a) Castledine et al. (2024) (b) Newbury et al. (2022)	5	(a) 60 cycles, ~400 gen. (b) 4 cycles, ~50 gen.	7 days	(a) 1/64 TSB	Soil compost	(a) Bacteria (b) Bacteria and phages
Mabisi inspired (proposed here)	5	24 cycles ~150 gen. (then longer)	3 days	Synthetic (MRS + M17 + YPD broth mixture) or milk (undefined)	Fermented milk	Bacteria and yeast (phages potentially)

**Box 1**

**Example of a synthetic community approach**

We here present a specific example model system to track contributions of species sorting and novel mutations on species and community level adaptation. In this example, we suggest a defined set of bacterial and yeast species taken from natural microbial communities of Mabisi—a traditionally fermented milk beverage from Zambia [55]; for example—*Lactobacillus helveticus, Lactococcus lactis, Acetobacter orientalis, Streptococcus thermophilus,* and *Geotrichum candidum.* Classical methods of microbiology (selective strain isolation, colony counts) would be combined with high throughput DNA sequencing and potential flow cytometry to track relative species abundances and identify new mutations in individual strains and species. We would allow for experiments over at least 500 bacterial generations to now include an evolutionary timescale. Taken together, this approach would be innovative by combining (1) using diverse species that represent a natural community, and (2) tracking both species sorting and genetic variant dynamics. It would open numerous experimental and theoretical research directions, such as (1) investigating repeatability of species sorting and genotypic changes concurrently following selection pressure through manipulating the environment with defined media or conditions (i.e. novel sugar, temperature, mixing), (2) mapping specific mutations and their roles in overall community performance, (3) linking community species profiles to community function in their metabolic output and resilience over time, and (4) comparing laboratory experimental evolution to the natural world (i.e. field experiments with cow milk).

**Potential work plan:**

1. *Mark co-existing strains (abundant species in Mabisi) with selectable markers and whole genome sequence each.* The markers, for instance an antibiotic resistance or unique ability to use certain growth compounds, would allow selective isolation from the community. This allows tracing their frequencies and isolating individual variants to track new mutations. The ability to selectively isolate species will allow testing evolved versus ancestor community members in various combinations.
2. *Co-culture the strains in a serial propagation experiment under two selective environmental conditions.* We would initiate replicate communities in a defined synthetic milk media (40 mL), grown at either 25°C or 37°C. These temperatures represent two distinct conditions known for their differing selective effect [56, 57]. Each replicate population would be serially transferred to fresh medium every three days and samples regularly frozen for later analysis. This would be repeated initially for 24 rounds of propagation, spanning just three months yet reaching ~160 generations of evolution. The experiment would ideally later continue for one year, reaching ~800 generations, or even longer.
3. *Map species sorting of the strains and fixation of novel mutations.* Relative species abundances at regular time points would be determined using 16S rRNA analysis, or machine learning models of flow cytometry data (absolute abundances also possible then). Selective plating with colony counts would support sequencing or flow cytometry results on relative species abundances (Fig. 1C). Additionally, profiles of entire communities at initial, middle, and end points would be completed through metagenomics to reveal population wide novel mutations; this would be validated by and combined with whole genome sequencing of random colony isolates (“individuals” of the community) to assign mutations to certain species.
4. *Selective plating and competition experiments.* Using selective plating, a library of evolved genotypes can be generated. These isolates would be used for competition experiments between evolved versus ancestor lineages in various combinations to measure the effects of new mutations on individual members, relative to within and between member species. This classical experimental evolution technique used extensively for single species systems, would be rare for multispecies communities.

Other model systems are emerging (Table 2) [21, 40, 41, 46–52], of which three we discuss here that are particularly related to our objective to detect both genotype and species sorting processes within a multi-species community over time—Cairns *et al*. (2018, 2020) [39, 53], Piccardi *et al*. (2019, 2022, 2024) [18, 42, 54], and Castledine *et al*. (2024) [19]. Cairns *et al*.’s experiments have impressively maintained a highly diverse community of over 30 species in laboratory cultures while detecting novel mutants in response to antibiotic pressure. Their ability to assess intraspecies mutations while tracking species level dynamics is an exciting and inspiring achievement that encourages our aspiration to explicitly trace genotypes within a species to create a multi-species Muller plot (Fig. 1B). However, a simpler community with fewer species, such as five proposed species from Mabisi, would benefit this goal. Neither Cairns *et al*.’s consortia choice of community members nor their artificially defined media reflect a specific natural environment, which can raise questions about translating findings to the real-world and applied questions. Furthermore, their time scale of 54 generations is commendable and sufficient under strong selection of antibiotic selection, but we argue longer propagation is desirable for investigation of evolutionary changes in milder novel environments. In comparison, Piccardi *et al*.’s experiments use a much simpler synthetic community of four species in an undefined metal working fluids media. Their four species used can be selectively plated and isolated from one another, which is advantageous for tracing genotypes to a particular species and double-checking the absence versus presence of types. A minor limitation is that bacterial growth in their metal working fluid is relatively slow at a 7-day transfer time. Despite slower growth, Piccardi *et al*.’s experiments have impressively reached 300 generations, which is a substantial period for predicted evolutionary dynamics from genotypic changes to arise. The undefined nature of metal working fluids can limit research questions regarding environmental selection pressures. An undefined media means that its resources (carbon, nitrogen, etc.) cannot be easily manipulated to test consequent effects on community traits, whereas an artificial and defined media opens more research directions. Nonetheless, Piccardi *et al*.’s use of a real-world substrate is valuable with applicable insight for bioremediation of metal working fluids. The five species bacterial community of Castledine *et al*. (2024) shares similarities with both our proposed system and Piccardi *et al*.’s system in that members are isolated by colony morphology and stably coexist for ~400 generations. Their experiments use a defined, and therefore potentially manipulatable laboratory media, but their community is not designed to mimic a natural soil community. They present a clear experimental approach to achieve a stable and reliable synthetic community using principles from traditional monoculture experimental evolution, such as invasion-from-rare and supernatant assays. Building upon existing model systems (Table 2), we propose a synthetic community inspired by fermented milk (Box 1), which stands apart through its representation of the natural community’s diversity and its unique potential to test community dynamics in both synthetic media and a natural environment (i.e. milk). We go on further to also discuss two specific questions in evolutionary ecology and how to experimentally investigate them with synthetic microbial communities.

Our proposed experimental system (Box 1) can be used to address an array of questions. For instance, **do more diverse communities follow more convergent or divergent paths?** There are two predictions of the ecological and evolutionary trajectories of communities in relation to their starting diversity. The first view is that more diverse communities are expected to have a limited number of potential evolutionary trajectories due to reduced available niche space. Less diverse communities in comparison should have more evolutionary space to explore and diverge from their original state, in an adaptive radiation process [58]. Conversely, the opposite may be true in the second view of “diversity begets diversity” [59, 60]; greater standing diversity is less restrictive and provides opportunity for divergence of lineages across replicate populations. An approach to explore this concept in depth would be to create synthetic microbial communities of isogenic clone combinations in varied levels of species and metabolic guild richness (i.e. capable of performing the same metabolic processes [61]) (Table 3), then track trajectories of community species compositions, genotype compositions, and metabolic profiles (i.e. function) over time. Predictions on whether diverse communities follow more convergent or divergent paths may differ depending on the biological level considered [62], so it is valuable to assess inter- and intra-species genetic diversity, as well as functions. The community compositions from metagenome sequencing could be compared between ancestor and propagated communities in the selection experiment where the initial diversity is varied. Variation among replicates and divergence from the ancestral population, both genetically and functionally (i.e. metabolic profiles) could then be compared between the different starting diversities.

**Table 3:** Suggested composition of synthetic communities to test community divergence in relation to initial community diversity.

**Initial composition**				**Initial diversity**	
**Focal species (lactic acid bacteria)**	**Other lactic acid bacteria**	**Acetic acid bacteria**	**Yeasts**	**Species richness**	**Guild richness**
*Lactobacillus helveticus*	*L. delbreuckii* *L. lactis*			3	1
*L. helveticus*		*Acetobacter orientalis* *A. pasteurianus*		3	2
*L. helveticus*	*L. delbreuckii* *L. lactis*	*A. orientalis* *A. pasteurianus*		5	2
*L. helveticus*	*L. delbreuckii* *L. lactis*	*A. orientalis* *A. pasteurianus*	*Geotrichum candidum*	6	3
*L. helveticus*	*L. delbreuckii* *L. lactis*		*G. candidum*	4	2
*L. helveticus*		*A. orientalis* *A. pasteurianus*	*G. candidum*	4	2

Starting communities would be created using isogenic clones of each species. Metabolic guilds in this case would be lactic acid bacteria, acetic acid bacteria, and yeasts.

Another research question that could be addressed is: **do evolutionary and ecological processes promote community stability to prevent invasion?** It is suggested that evolutionary processes can contribute to stability at the community level in species composition and function [1, 63]. For example, if true, it is expected that communities initially unable to resist invasion will later exhibit increased stability after undergoing experimental evolution. Specialization, generalization, and diversification are evolutionary trajectories that can create complementarity in resource use or overall niche space to enhance exclusion of an invader [64]. Simply ecological processes (i.e. species sorting) could also independently increase stability against a novel species establishing since shifts in relative species abundances without genotype changes could improve total resource use and leave less available niche space. Long-term propagation of a synthetic microbial community could test this by (a) first, adjusting the initial diversity by removing one or more members, therefore predictably opening ecological niche space for a potential invader, (b) propagating communities with or without an invading species for hundreds of generations [65], and (c) the invading species would be marked (i.e. antibiotic resistance) for selective plating isolation at multiple time points, therefore allowing two by two factorial invasion tests of naïve versus evolved invader into naïve versus evolved resident communities. Stability, or resistance, would be measured as the inverse of invader abundance—determined simply by cell counts on selective agar plates, or alternatively by relative abundances through 16S rRNA gene sequencing.

In summary, experimental approaches with a defined microbial community extend concepts from classical experimental evolution of single species to study the complexities of community level changes. Successfully assembling a microbial community, with traceable members, in a defined and manipulatable environment, would provide an effective opportunity to experimentally study general processes and principles in evolutionary ecology. The dynamics between species sorting and genotypic changes is a core, yet not well understood, topic across biological research [3, 4]. The use of a synthetic microbial community inspired on natural communities reproduced in laboratory environments (e.g. fermented foods), enables for experimental evolution of diverse communities. Appreciating the capabilities of communities to respond to changing environments, and consequent selection pressures beyond genetic changes within single species, must be understood in the face of rapid global environmental changes. Investigating the dynamics between intra- and interspecies processes is significant for understanding community responses to change in natural and managed systems across short and longer temporal scales.

## Data Availability

Data sharing not applicable, since no datasets were generated or analysed.
